# Tomato Brown Rugose Fruit Virus: Survival and Disinfection Efficacy on Common Glasshouse Surfaces

**DOI:** 10.3390/v15102076

**Published:** 2023-10-11

**Authors:** Anna Skelton, Leanne Frew, Richard Ward, Rachel Hodgson, Stephen Forde, Sam McDonough, Gemma Webster, Kiera Chisnall, Mary Mynett, Adam Buxton-Kirk, Aimee R. Fowkes, Rebecca Weekes, Adrian Fox

**Affiliations:** Fera Science Limited, York Biotech Campus, York YO41 1LZ, UK; leanne.frew@fera.co.uk (L.F.); richard.ward@fera.co.uk (R.W.); stephen.forde@fera.co.uk (S.F.); sam.mcdonough@fera.co.uk (S.M.); gemma.webster@fera.co.uk (G.W.); kiera.chisnall@fera.co.uk (K.C.); mary.mynett@fera.co.uk (M.M.); adam.buxton-kirk@apha.gov.uk (A.B.-K.); aimee.fowkes@fera.co.uk (A.R.F.); rebecca.weekes@fera.co.uk (R.W.); adrian.fox@fera.co.uk (A.F.)

**Keywords:** tomato brown rugose fruit virus, ToBRFV, disinfection, survival, thermal inactivation

## Abstract

Tomato brown rugose fruit virus (ToBRFV) is a contact-transmitted tobamovirus affecting many tomato growing regions of the world. This study investigated the effects of different glasshouse surfaces on the survival of the virus; the efficacy of different disinfectants; and heat treatment against ToBRFV (surfaces included steel, aluminium, hard plastic, polythene, glass and concrete). A bioassay followed by ELISA was used to check virus viability. ToBRFV survived for at least 7 days on all surfaces tested and on some for at least 6 months. The virus survived for over two hours on hands and gloves. Hand washing was shown to be unreliable for the removal of the virus. Glutaraldehyde and quaternary ammonium compound disinfectants were effective at one hour on all surfaces. Some other disinfectants were effective at one hour of contact time, on all surfaces except concrete. Sodium hypochlorite was partially effective against ToBRFV, even on concrete. A 5 min soak of plastic trays in water at 90 °C was effective at denaturing ToBRFV; however, 5 min at 70 °C was not. Heating infected sap showed the thermal inactivation point to be 90 °C, confirming the hot water treatment results and showing that deactivation was due to the heat treatment and not a washing effect of the water.

## 1. Introduction

Tomato brown rugose fruit virus (ToBRFV) is an emerging viral pathogen that has been reported infecting tomatoes throughout Europe, North America, China and the Middle East [1,2]. Tomato brown rugose fruit virus is a species in the genus *Tobamovirus*, family *Virgaviridae*. As with other members of the genus, the virus is expected to have virions that are thermally stable and survive for long periods of time in plant sap [3]. The virus can overcome the *Tm*-2^2^ resistance gene that confers varietal resistance to other tobamoviruses, such as tobacco mosaic virus (TMV) and tomato mosaic virus (ToMV) [2]. The virus is a major threat to global tomato production due to both yield loss and fruit symptoms, leading to unmarketable fruit due to marbling and distortion [4,5]. The virus has also been detected from natural infections of pepper (*Capsicum annuum*) [6].

Given the threat that the virus presents to tomato production worldwide, the virus has become the subject of regulatory controls [1,7]. The key areas where research has been focused are understanding the epidemiology of the virus and developing improved diagnostic methods for the detection of the virus during outbreaks and from seed in trade. Understanding the epidemiology of a disease is essential to understand and predict the dynamics of disease development and decide on which (and when) mitigations should be taken to prevent further spread or limit the impact of the disease [8]. The virus can spread rapidly through a susceptible crop, through normal working practices [9]. Understanding routes of entry into glasshouse crops and the long-distance dissemination of the virus is more challenging. The virus has been shown to be seed-borne on the seed coat of tomato, but not carried within the seed [10,11]. Due to the risks associated with seeds and the need for early detection to mitigate spread, there have been multiple reports of new serological and molecular diagnostic assays [2]. The result of these rapid diagnostic developments has also led to the development of internationally recognised diagnostic standards for the detection of the virus in both plants and seeds [6,12]. Additionally, the increased use of high-throughput sequencing in plant virology laboratories has enabled the analysis of multiple full-length genomes through a Nextstrain build, giving further information on the origins and diversity of this emerging virus [13,14,15]. 

There has been less focus on the practical aspects of outbreak management, such as understanding how resilient ToBRFV is with regard to natural degradation, and also the efficacy of disinfectants at reducing the risk of carry-over infection through prophylactic use or re-infection after an outbreak. Samarah et al. [16] reported that contaminated seed could be disinfected with 30 min exposure to 2% hydrochloric acid or 3 h exposure to 10% trisodium phosphate solution. Chanda et al. [17] investigated the efficacy of a range of disinfectants against ToBRFV and cucumber green mottle mosaic virus (CGMMV; genus *Tobamovirus*) and found that some of these were effective against liquid inoculum preparations over short exposure times; however, this work did not study the potential impact of surface substrates on the efficacy of disinfectant regimes. It has previously been demonstrated that the substrate surface can influence the survival of contact-transmissible viral pathogens. For example, potato spindle tuber viroid (PSTVd, genus *Pospiviroid*) has been shown to remain infectious for a greater length of time on leather or string than on surfaces such as plastic or metal [18]. There are a wide range of surfaces found in a commercial glasshouse environment, which range from the presumed inert (e.g., glass and stainless steel) through to porous surfaces such as concrete and wood. Additionally, there are a wide range of plastics used within glasshouse operations, ranging from polythene sheeting to hard plastic picking crates, which may also impact the disinfection efficacy. These surfaces may all impact both the survival of a pathogen and the efficacy of disinfection. Following an outbreak, these would all need to be sanitised before a following crop is planted to avoid the risk of carry-over infection, as some of this equipment (e.g., picking crates or plant trollies) may circulate between glasshouses on a specific site or may even transfer between different commercial operations [2]. Whilst the work of Ehlers et al. [19,20] focuses on shoes and clothing, there have, thus far, not been any specific studies looking at the influence of these glasshouse surfaces on the survival of, or on the efficacy of disinfection against, ToBRFV. 

As a member of the genus *Tobamovirus*, it would be expected that ToBRFV would be robust, with long retention of infectivity (survival) and resistance to disinfection by comparison to other viral pathogens outside of this genus. It is essential to identify effective routine disinfection regimes for prophylactic hygiene treatments. Therefore, this study reports a series of experiments that aimed to determine the survival of ToBRFV and the efficacy of disinfection approaches on a range of surfaces commonly found in a glasshouse environment, including human skin, steel, aluminium, hard plastic, polythene, glass and concrete, with disinfection approaches including thermal deactivation and a range of chemical active ingredients. 

## 2. Methods

The aim of the work described here was to ascertain the viable survival time and determine disinfection efficacy, including thermal inactivation, for ToBRFV. This included a range of experiments to determine survival on a range of surfaces, including hands, gloves, concrete, glass, steel, aluminium, hard plastic and polythene. The efficacy of disinfectants was determined on each of these surfaces. Thermal inactivation was determined both by soaking contaminated plastic surfaces in hot water and confirmed by heating sap from infected plants. The infectivity of sap residues was measured by a bioassay and confirmed via ELISA.

### 2.1. General Experimental Set-Up

ToBRFV (isolate PC-1236, DSMZ) was inoculated onto tomato plants, cv. Moneymaker. Leaf material was collected two to three weeks after inoculation and confirmed positive by real time RT-PCR using the specific ToBRFV assay of Menzel and Winter [6,21], amended with iTaq universal probes one-step reaction mix (Bio-Rad). The cycle threshold (Ct) values ranged from 10 to 12. The infected leaves were ground in water (1:5 dilution) and approximately 200 µL sap was used to contaminate an area of approximately 20 cm^2^ on a range of different surfaces (glass microscope slide, concrete block, aluminium sheet, hard plastic glasshouse tray, polythene sheeting, stainless steel bar, hands and nitrile gloves), by using a cotton bud to produce a thin visible layer of sap. In additional experiments (hand washing and survival on hands and gloves), the surfaces were also contaminated by lightly rubbing with infected leaves from the same source plants. To account for potential differences in hand surfaces and washing techniques, two people of different ages, one male and one female, were selected to carry out the experiments. 

### 2.2. Bioassay for Determination of Viable Virus

For both the survival and disinfection experiments, virus viability was demonstrated by taking swabs at various time points and testing by bioassay and ELISA. Once the inoculum was dry, swabs were taken after initial contamination to show that the initial inoculum was viable. In the case of survival studies, further swabs were taken at specified time points. In the case of hand washing, disinfection and heat treatment studies, swabs were taken post-treatment as specified below. At the end of setting up each experiment, a swab was taken from the buffer and inoculated onto test plants, as a control, to show that the buffer had not become contaminated.

Cotton buds, soaked in phosphate buffer pH7 containing celite (Sigma-Aldrich, Gillingham, UK), a mild abrasive powder, were used to take swabs from different surfaces. Swabs were taken by rubbing the surface with the cotton bud and then these were gently rubbed onto the leaves of *Nicotiana tabacum* plants cv. White Burley (approximately 5 weeks from sowing). The inoculated leaves were marked by a hole from a pipette tip prior to inoculation. Gloves were worn and changed between each replicate. The *N. tabacum* plants were then covered with a bread bag (Cater for You, High Wycombe, UK), placed on individual plastic saucers to avoid cross-contamination and placed in a glasshouse at 20 to 25 °C for 2 to 3 weeks. *N. tabacum* was used as a test plant as it is susceptible to ToBRFV and rapidly shows symptoms on the inoculated leaves.

Following visual assessment, the inoculated leaves were removed and tested by ELISA for ToBRFV using antisera from DSMZ (Braunschweig, Germany) according to the manufacturers’ instructions. An uninfected (healthy) *N. tabacum* leaf was used as a negative control. Samples with absorbance values greater than three times the negative control’s absorbance value were classified as positive. ELISA was performed to confirm that any lesions seen were due to the virus and not from mechanical damage or phytotoxic effects of the disinfectants. 

For each variable, e.g., surface and time, three swabs were taken, and each inoculated onto an individual test plant to give three repetitions for each sample point. All experiments were performed in duplicate at different time points to determine whether results could be consistently generated.

### 2.3. Survival on Skin and Gloves

Survival was investigated by exposing skin and gloved hands (nitrile-type disposable gloves) to infected sap, and secondly by rubbing the hands with infected leaves. The bare hand and gloved hand were swabbed at 15 min intervals up to 1 h and then 30 min intervals up to 2 h. 

### 2.4. Survival on Glasshouse Surfaces

A range of glasshouse surfaces (glass, concrete, aluminium, hard plastic, polythene and stainless steel) were surface-contaminated with ToBRFV-infected leaf sap. A picking crate from a tomato grower was used as the hard plastic surface. The surfaces were kept at ambient temperature throughout the trial (approximately 20 °C) and swabs were taken at different time periods (ranging from 2 h to 6 months) and inoculated onto test plants, to ascertain the length of time for which the virus survived on different surfaces. 

### 2.5. Hand Washing to Reduce Contamination Risk

Hands were rubbed for approximately 5 s with leaves from ToBRFV-infected tomato plants. Hands were then washed for 30 s or 1 min using the following washes: water only, water and household soap, water and medicated hand wash (Hibiscrub^®^, Mölnlycke, Milton Keynes, UK) and water and medicated hand wash followed by an alcohol gel (Purell, Milton Keynes, UK, 70% ethyl alcohol).

### 2.6. Efficacy of Hot Water Treatment Combined with Disinfection

Sections of a hard plastic glasshouse tray were contaminated with ToBRFV-infected sap and left to dry. Pre-treatment swabs were taken from the tray sections and inoculated onto healthy test plants to show that the virus on the trays was infectious. The tray sections were then soaked in hot water at either 70 °C or 90 °C for 5 min. After soaking, swabs were taken and inoculated onto test plants and then the tray sections were sprayed with 1% Virkon S (the recommended rate) (Scientific Laboratory Supplies, Nottingham, UK) and left for 1 min, before more swabs were taken and inoculated onto test plants. 

### 2.7. Thermal Inactivation

To investigate whether any inactivation of the virus, after hot water treatment, was due to the heat treatment or also a washing effect of the water, ground ToBRFV-infected tomato sap (1:10 dilution in water, 500 µL) was placed in 1.5 mL micro-centrifuge tubes (Starlab, Milton Keynes, UK) suspended in a water bath, for 5 min, at various temperatures ranging from 70 °C to 95 °C; 70 °C is the temperature used for pasteurisation and 90 °C is the thermal deactivation temperature for cucumber green mottle mosaic virus (CGMMV) [22]. After incubation, a cotton bud was dipped in each tube and then rubbed onto test plants to check for transmissibility. After 2 weeks, the test plants were tested by ELISA for the presence of ToBRFV.

### 2.8. Efficacy of Disinfection Approaches

As with the survival on glasshouse surfaces experiment, the six surfaces were contaminated with ToBRFV-infected leaf sap. Once the sap on the surfaces was dry, swabs were taken from the surfaces and inoculated onto test plants, to show that the virus was infectious. The surfaces were then sprayed with a disinfectant using a domestic spray bottle (SLS, Nottingham, UK), at the recommended rate, and left for various contact times before swabs were taken and inoculated onto test plants. The disinfectants included in the study were chosen because they are widely available to growers and cover several different groups of active compounds. Menno Florades (Royal Brinkman, Woodmansey, UK) was chosen as it is approved for use as a plant protection product in the European Union (Table 1). Huwa-San (Roam Technology, UK) was used at both 3% and 12.5% active ingredient. The 12.5% a.i. is the rate recommended for fogging and the 3% a.i. for surface disinfection. Menno Florades was applied as a foam using a proprietary foaming applicator (MENNO Chemie GMBH, Norderstedt, Germany).

Initially, a 1 min contact time was investigated for several disinfectants (Menno Florades, Jet 5 (Certis, Cambridge, UK), sodium hypochlorite and Virkon S)); however, as this short contact time was ineffective, a 60 min contact time was used for all the disinfectants. Minimum effective contact times were then investigated for the disinfectants that showed the best results at 60 min.

### 2.9. Molecular Detection of ToBRFV Post-Disinfection Treatments

Swabs were taken from different surfaces contaminated with ToBRFV-infected sap before and after being sprayed with Virkon and Unifect G (Aromany, Bridgwater, UK) (see Table 1 for the rates). The surfaces (glass, concrete, aluminium, hard plastic, polythene and stainless steel) were swabbed 10 min after application of the disinfectant. Swabs were placed in 1 mL of phosphate buffer in a 50 mL universal tube. The nucleic acid was then extracted from the swab buffer using an RNeasy plant mini kit (Qiagen, Manchester, UK) and tested by real-time RT-PCR using the specific ToBRFV assay of Menzel and Winter [6,21]. Swabs were also taken from the surfaces before and after disinfection and inoculated onto *N. tabacum* test plants, to check for viability.

## 3. Results

### 3.1. Survival on Different Surfaces

The results indicate the length of time that sap containing ToBRFV remains viable on a range of surfaces commonly found in a glasshouse, including surfaces representing structural components (aluminium, glass, concrete), equipment and surfaces encountered in routine work (hard plastic, polythene sheeting, stainless steel, disposable gloves) and also human skin (Table 2). The positive test results presented for human skin and gloves represent the final sampling time point of two hours (Table 2). Both skin and gloves were contaminated with leaf sap, or via leaf rubbing, and then sampled and tested every 15 min for two hours and were consistently positive for infectious virus throughout the sampling period (interim time point data for skin and gloves presented in Supplementary Material Table S1). 

ToBRFV remained viable on all glasshouse surfaces after seven days, infecting all test plants in both experimental repetitions. From 28 days post-contamination, in some cases, there was a difference in the number of positive plants between the two repetitions of the experiments. The virus was still active on most surfaces after 90 days, except for aluminium. Results for concrete were inconsistent: it was found to be negative for ToBRFV after 28 days but positive again after 90 days. ToBRFV was still infectious in sap swabs from glass, hard plastic and polythene even after 180 days, although not all plants were infected in both trial repetitions, indicating some drop in infectivity. Overall, the results show that there is a good chance that ToBRFV will survive on surfaces for (at least) 28 days, and on some surfaces for at least 6 months. 

### 3.2. Efficacy of Hand Washing for Removal of ToBRFV Inoculum

The results from investigations on the efficacy of hand washing at removing ToBRFV from hands are presented (Table 3). Washing for only 30 s was largely ineffective. Washing for 30 s with water alone had no apparent effect on the presence of infectious ToBRFV on the hands. Washing for 30 s with other treatment combinations including soap, medicated hand wash (Hibiscrub^®^, York, UK) and hand wash followed by antibacterial gel all appeared to have some limited effect on reducing the levels of infectious ToBRFV on the hands. 

Washing contaminated hands for 1 min in water alone, water plus household soap and water with Hibiscrub^®^ plus an antibacterial hand gel appeared successful at removing ToBRFV. Water plus Hibiscrub^®^ was only partially successful at 1 min. 

### 3.3. Efficacy of Hot Water Treatment Combined with Disinfection

Table 4 shows that soaking plastic trays in water at 70 °C was ineffective at deactivating ToBRFV at this temperature; however, 0% infection was seen at 70 °C if the trays were then sprayed with 1% Virkon after the soak. Soaking the trays at 90 °C was effective with or without the 1% Virkon spray.

### 3.4. Thermal Inactivation

The results presented in Table 5 show that whilst each increase in temperature did reduce the number of lesions seen on each test plant, plants with no lesions were only seen with soaking at 90 °C and 95 °C at 5 min. Moreover, 85 °C partially worked as the number of lesions were reduced after treatment but a 100% reduction was not achieved. This suggests that 90 °C is the temperature required to deactivate ToBRFV.

### 3.5. Efficacy of Disinfection Approaches

All the disinfectants tested (Menno Florades, Jet 5, sodium hypochlorite and Virkon) at a 1 min exposure time failed to give control of ToBRFV (Supplementary Material Table S2). Subsequent trials of disinfectants focused on a 60 min exposure. 

Unifect G and Virocid (CID lines, UK) (the glutaraldehyde and quaternary ammonium compound disinfectants) were the most effective on all surfaces at deactivating the virus. These glutaraldehyde/quaternary ammonium products and sodium hypochlorite were the only products to be effective on concrete consistently. Even the increased concentration of Huwa-San (active ingredient, hydrogen peroxide), which was effective on all other surfaces, was not effective on concrete. Differing results were seen for Virkon S (a.i. potassium peroxymonosulfate) on concrete, with the first experiment giving 100% effectiveness and the repeat experiment showing two out of three plants infected post-treatment. Virkon S was effective on all other surfaces with a 1 h contact time.

Menno Florades (a.i. Benzoic acid) was mostly effective on all surfaces except concrete. Sodium hypochlorite is partially effective at denaturing ToBRFV on polythene, glass and stainless steel and is completely effective against ToBRFV on other surfaces. Huwa-San (3% a.i.), Jet 5 (a.i. peroxyacetic acid) and trisodium orthophosphate (TSOP) were not very effective against ToBRFV with a 1 h treatment. 

Hard plastic and polythene were potentially the surfaces that the disinfectants deactivated ToBRFV on most successfully. Stainless steel, aluminium and glass were varied in their results, with all disinfectants showing a general reduction in infectivity. Concrete was the surface where the disinfectants had the least effect. Overall results show that Unifect G and Virocid, disinfectants containing glutaraldehyde and quaternary ammonium compounds as the active ingredients, reduced the infectivity of ToBRFV the most after 60 min, with Virkon S (potassium peroxymonosulfate), Menno Florades (a.i. benzoic acid) and a high concentration of Huwa-San (12.5% a.i.) (a.i. hydrogen peroxide) also resulting in a large reduction in infectivity. However, on all surfaces, there was only a small reduction in ToBRFV infectivity after treatment with Huwa-San at 3%.

Treatment with Unifect G, for one hour, was shown to be 100% effective against ToBRFV on all surfaces (Table 6), and further testing showed that it also had the same effect after just 10 min of treatment (Table 7). At 10 min contact time, Virkon S was only partially effective; however, on many of the test plants, only one lesion was seen, suggesting that a longer contact time would be effective. This is supported by the results for 20 min contact time, where every surface except concrete saw a 100% reduction in ToBRFV infectivity. However, the Virkon S results for concrete were very variable as a 10 min contact time gave 100% effectiveness, 20 min contact time showed no effect against ToBRFV infectivity and 1 h contact time gave differing results for each repeat of the experiment. ToBRFV was still active on concrete even after a 16 h contact time with Menno Florades (benzoic acid) and Huwa-San 3% a.i. (hydrogen peroxide), suggesting that concrete may be a challenging surface to disinfect. Polythene, stainless steel and hard plastic all returned similar results for Huwa-San 3% (a.i. hydrogen peroxide), which failed to completely inactivate ToBRFV even after 16 h. It was shown to be partially effective at deactivating ToBRFV on aluminium and glass after 16 h, more effective than at 60 min but not 100% effective. Overall, Menno Florades (disinfectant with active ingredient benzoic acid) at a 16 h contact time and Virkon S (a.i. potassium peroxymonosulfate) at a 20 min contact time were 100% effective on every surface except concrete, and Virocid (60 min) and Unifect G (10 min) (a.i. glutaraldehyde and quaternary ammonium compounds) were effective on all surfaces.

### 3.6. Molecular Detection of ToBRFV Post-Disinfection Treatments

All indicator plants inoculated from surfaces after treatment with Unifect G, after 10 min contact time, were negative by ELISA for ToBRFV. However, all swabs taken directly from the surfaces were positive by real-time RT-PCR (Table 8). Whilst the Ct values were higher post-treatment than pre-treatment, suggesting a reduction in viral concentration, they were still low, indicating a strong positive result. Real-time PCR does not distinguish between viable and nonviable virus, whereas a bioassay followed by ELISA detects viable virus. Aluminium, hard plastic and stainless steel were positive by both ELISA and real-time RT-PCR both pre-treatment and post-treatment with Virkon. Alternatively, glass, polythene and concrete were negative by ELISA post-treatment with Virkon but were positive by real-time RT-PCR. The CT values do not appear to be notably affected by the addition of Virkon; in fact, in some instances, the Ct values appeared to be slightly lower post-treatment with Virkon than they were pre-treatment.

## 4. Discussion

There have been a limited number of studies published on the efficacy of disinfectant treatments. Two of these studies have focused on seed treatments to reduce the risk of entry to a production system [10,16]. A third study [23] investigated the disinfection of ToBRFV-contaminated soil. Chanda et al. [17] reported the efficacy of disinfectant treatments; however, they focused on the rapidity of virus inactivation using disinfectants mixed with infected sap. There have also been studies conducted on the efficacy of the disinfection of clothing and shoes for the eradication of the virus [19,20]. Due to the expected robust, thermally stable nature of tobamoviruses and the ease with which they can be mechanically transmitted, it is important to determine effective ways to inactivate ToBRFV, with respect to common surfaces. Post-outbreak contamination of these surfaces would be expected and effective ways to manage the virus and reduce the risk of environmental contamination need to be determined. Therefore, the aims of the study were to determine the survivability of ToBRFV on common surfaces and touchpoints in a glasshouse/nursery and the efficacy of commercially available disinfectants on these surfaces. This information could then be used to support strategies for virus management to minimise the risk of outbreaks and to inform post-outbreak disinfection procedures. In brief, this study reports the duration for which the virus remains infectious on a range of surfaces commonly found in a glasshouse environment, including glass, concrete, aluminium, stainless steel, hard plastic and polythene (soft plastic). The study also reports the efficacy of a range of disinfection strategies for the treatment of these surfaces, including thermal and chemical approaches. It also shows that ToBRFV is a highly stable virus and can remain viable on some surfaces for at least 6 months. The virus is thermally stable, remaining infectious following both hot water treatment and in heated sap, at up to 90 °C for 5 min. From this study, several products have been identified as being effective at inactivating ToBRFV across a range of surfaces; however, these data indicate that the virus may still be detected by molecular diagnostic methods post-disinfection even when the virus is no longer infectious. This is the first study to look at the influence of different glasshouse surfaces on the survival of ToBRFV and the efficacy of disinfection against this virus.

### 4.1. Survival on Hands and Hand Washing

ToBRFV will remain infectious on both skin and disposable (nitrile-type) gloves for at least two hours. Tomato plants are repeatedly handled throughout their production cycle for side-shoot removal, stringing, fruit picking and other activities. The length of survival of the virus underlines the potential for the virus to rapidly spread through a crop via normal production practices. Additionally, the length of time in which the virus can contaminate hands highlights the need for effective biosecurity measures on entry to the production facility. Hand washing results were very variable, even when repeating the same washing conditions, suggesting that it may be the physical act of washing the hands that removes the majority of the virus and not the chemicals used. However, this may also be due to different levels of the virus being deposited on the hands from rubbing infected leaves, or different hand washing techniques used by individuals. As hand washing is unreliable, to achieve the thorough elimination of the virus, a wash of more than 30 s is required, which would be difficult to apply and enforce in practice. In practice, the worker’s hands would be highly contaminated and a 30 s or 1 min hand wash would be unlikely to eliminate the ‘green sap’ from hands. The wearing of gloves and changing them as often as needed would eliminate the need to monitor hand washing times. These hand washing results were consistent with the findings of Chanda et al. [17], who found alcohol-based chemicals such as hand gel to be ineffective in deactivating ToBRFV.

### 4.2. Survival on Glasshouse Surfaces

The survival results presented here demonstrate that the virus remains infectious on some glasshouse surfaces as a potential inoculum for at least 6 months. These data also indicate that the survival rate of the virus is dependent on the surface on which it is present. This difference in survival between different surfaces is similar to the results found by Mackie et al. [18] looking at the surface survival of PSTVd. These different survival times are further evidence that a general overarching cleaning regime may not be as effective as a targeted approach for each surface, depending on the survival characteristics of each pathogen on each surface; for example, concrete gave inconsistent results in both the survival and disinfection experiments. This may be due to the rough surface on the concrete, or because it is more porous than the other surfaces, making it more difficult to remove the virus using the swab.

### 4.3. Disinfection Efficacy 

Effective disinfection regimes are essential for post-outbreak clean-up as well as for prophylactic routine disinfection. The efficacy of several different disinfectants was evaluated on a range of surfaces representative of those commonly found in a glasshouse or nursery. The products included in the study were chosen because they are widely available to growers and cover several different groups of active compounds (Table 1). These disinfectants were evaluated at manufacturers’ recommended concentrations for use and to establish a minimum exposure time for these products. Initially, all products were trialled at one-minute exposure, and all the products tested failed to inactivate ToBRFV at this short contact time (Supplementary Material Table S2). All products were therefore trialled at one hour and Menno Florades, Huwa-San (12.5% a.i.) and Virkon S had at least moderate efficacy against ToBRFV on most surfaces, except concrete. Menno Florades is effective at a 16 h contact time except on concrete. Similar results were seen by Ehlers et al. [20], who showed that Menno Florades was nearly completely effective at the inactivation of ToBRFV in foot disinfection mats after 24 h, and, after 4 days, it achieved the complete inactivation of ToBRFV. Ehlers et al. [19] found Menno Florades to be highly effective at the removal of infectious ToBRFV from contaminated fabrics and also the inactivation of the virus from contaminated cleaning solutions. Menno Florades is the only disinfectant tested that is approved for use as a plant protection product in the European Union. Ehlers et al. [20] discussed the need for a standardised method for testing. The differences in the results from this study and Chanda et al. [17] further emphasise the need for this. 

Sodium hypochlorite is partially or completely effective at denaturing ToBRFV depending on the surface. This active ingredient also reduces seed-borne virus transmission: Davino et al. [10] found 2.5% sodium hypochlorite, at a 15 min contact time, to be 100% effective against ToBRFV-contaminated seed. The results in the present study are consistent with Chanda et al. [17], who observed that 10% Clorox (a.i. sodium hypochlorite) reduced ToBRFV infection in a sap solution. Rodríguez-Díaz et al. [24] also found 3% sodium hypochlorite to give a 99% reduction in lesions in bioassay tests.

Huwa-San, at the concentration recommended for surface disinfection, was ineffective against ToBRFV at the recommended contact time. At the concentration recommended for use in large nebulising systems (cold foggers), it was effective against ToBRFV at 1 h, except on concrete. Davino et al. [10] found that hydrogen peroxide was not effective on contaminated seed, with 100% positive results by RT-qPCR; however, the samples all tested negative by bioassay. Virkon S was effective after 20 min contact time at deactivating ToBRFV, except on concrete. These results are similar to those found by Chanda et al. [17], who showed Virkon S to be mostly effective after only 60 s in infected sap at 3% and partially effective at 2%; however, these are higher concentrations of Virkon S than used in this study, where 1% Virkon S was used. This suggests that whilst an increase in concentration could make the deactivation time of ToBRFV shorter, manufacturer guidelines recommend only using 1% Virkon S for routine use on surfaces (https://nrm.co.nz/wp-content/uploads/2017/09/Virkon-S-UsageGuide.pdf, accessed on 13th September 2023). The effectiveness of 1% Virocid at the deactivation of ToBRFV matches the findings of Chanda et al. [17], who found that 0.5% and 2% Virocid resulted in total inactivation after only 60 s of mixing the disinfectant with infected sap. This suggests that Virocid can be effective at low concentrations and short exposure times. The similar results for Unifect G are likely to be due to the same active ingredients, glutaraldehyde and quaternary ammonium compounds.

In this study, trisodium orthophosphate was not totally effective against ToBRFV on any of the surfaces. Chanda et al. [17] found similar results, with most replicates testing positive but at much shorter contact times (60 s of mixing trisodium phosphate with infected sap). However, the results from Davino et al. [10] showed trisodium phosphate to be mostly effective, when seeds were submerged for 180 min, suggesting that longer exposure is needed for this a.i. 

### 4.4. Thermal Inactivation

Soaking contaminated tray sections in hot water at 90 °C for 5 min deactivated the virus, but 70 °C was not effective, which is consistent with findings on other tobamoviruses; for example, CGMMV in sap is inactivated by 10 min at 90 °C [22]. Hot water treatment was used as a small-scale methodology to test temperature effects on ToBRFV. Commercially, plastic trays are now being steamed by some growers at 95 °C for approximately 40 min. The results from the heat treatment of infected sap confirm the previous hot water treatment results and show that the inactivation is due to the heat treatment. 

### 4.5. Molecular Detection of ToBRFV Post-Disinfection

The results from testing swabs by real-time RT- PCR show that there are issues when using this method for the detection of ToBRFV post-disinfection. Viral RNA that does not appear to be biologically active was found to still be detectable, by real-time RT-PCR. Swab testing can provide extra assurance to growers that the virus is absent and as a management tool, giving early warning of an outbreak and determining how far the virus has spread within a glasshouse. However, due to ToBRFV detection after disinfection, despite inactivation, it is not recommended that swab testing by PCR is carried out after crop clean-up or to declare eradication. If swab testing is carried out in the event of an outbreak after clean-up, it would be recommended to also have swabs tested by inoculation onto test plants to give more confidence in the clean-up procedure.

### 4.6. Limitations of the Study

It must be noted that ground infected sap was added to each surface, and this may be an artificially high amount of virus. Moreover, the surfaces were not washed before disinfection, which would physically remove some of the virus. The disinfectants tested were all used at the recommended rates, except Huwa-San (12.5% a.i.), but were not all used at the recommended contact times, as the aim was to find a contact time that was useful in as many situations as possible. An issue that arose briefly during this experiment was the inconsistency of the positive controls showing virus symptoms on indicator plants. During the disinfection of contaminated surfaces experiment, the positive controls, which were indicator plants inoculated with swabs taken from the different ToBRFV-contaminated surfaces, before the surfaces were sprayed with disinfectant, did not show clear virus symptoms. As the virus had previously been shown to survive on all surfaces for at least 7 days and up to 6 months, it was very unusual that the controls were not positive after less than an hour on each surface. The most likely explanation for this is the light levels in the glasshouse where the test plants were kept after inoculation. These test plants were kept in the glasshouse with LED lights in December, when the general light levels were very low. The International Seed Federation protocol on the detection of ToBRFV in seed [12] recommends at least 12 h of light for inoculated test plants. These plants did receive 12 h of light, but the LED lights may not have given a suitable light level. In subsequent re-testing, metal halide growth lights were used and symptoms were as expected. Other laboratories have found issues with LED lights and virus symptoms not being seen (Anne Giesbers, NIVIP, The Netherlands, personal communication). Whilst some disinfection strategies reduced the inoculum of the virus on some surfaces, given the long survival times of this damaging virus, the aim should be to follow strategies that eradicate the virus to prevent carry-over infection to future crops. 

### 4.7. Further Work and Summary

It would be of interest to look at other time points for the disinfection work and to determine other methods to establish the difference between detection and viability to avoid having to perform extensive bioassays.

ToBRFV is very stable, but there are possible ways to denature the virus to prevent it from spreading and infecting plants. Several products had success across multiple surfaces, the most difficult surface being concrete, where greater care to disinfect should be taken. Gloves are recommended alongside hand washing, to control both the spread of viruses such as ToBRFV and other contact transmitted pathogens. Hot water treatment seems sufficient for plastic trays. While denaturing of the virus is possible, it is likely that ToBRFV detection will persist, as swabs of disinfected surfaces can still give positive results when tested by real-time RT-PCR. Therefore, real-time RT-PCR is not suitable to indicate the eradication of ToBRFV following an outbreak, due to persisting molecular detection. These studies here take no account of the “food-safe” aspects of the disinfectants and growers should consider potential residues when formulating disinfection strategies. The extended contact times required for the efficacy of some of the disinfectants investigated in this study will provide practical challenges to growers in implementing effective disinfection regimes. However, the focus of cleaning and disinfection protocols should be the eradication of this virus, as well as other contact-transmitted pathogens, such as PSTVd. At present, there are no studies on overarching disinfection protocols, and it would be useful for future work to consider combination treatments for the range of potential pathogen threats in the tomato glasshouse.

## Figures and Tables

**Table 1:** Disinfectants tested against ToBRFV inoculum. Products are listed under their commercial name (brand), active ingredient, concentration in formulation (where known), dilution rate in trial and final concentration. (N/A) denotes concentrations not known.

Product	Active Ingredient (a.i.)	% Active in Formulated Product	Product Dilution Used for Trial	% Active
Virkon S	Potassium peroxymonosulfate		1 tablet in 500 mL water	1%
Menno Florades	Benzoic acid	9%	4% applied as a foam	0.36%
Jet 5	Peroxyacetic acid	5%	1:125	0.04%
Huwa-San TR 50	Hydrogen peroxide	50%	25%	12.5%
Huwa-San TR 50	Hydrogen peroxide	50%	6%	3%
TSOP	Trisodium orthophosphate		10%	10%
Sodium hypochlorite	Sodium hypochlorite	Approximately 1%	20 mL in 500 mL water	0.04%
Unifect G	Glutaraldehyde and quaternary ammonium compounds	N/A	1:25	N/A
Virocid	Glutaraldehyde and quaternary ammonium compounds	N/A	1%	N/A

**Table 2:** ELISA results of test plants inoculated with swabs taken from surfaces contaminated with ToBRFV-infected sap at different time periods. Each result is from two repetitions of three plants per repetition. + = all plants positive in both repetitions, − = all plants negative in both repetitions. n, n = numbers are where infection was incomplete and reflect the number of plants positive out of three in each repetition of the experiment, N/A = time point not tested. * Longest time point only shown; see Supplementary Material Table S1 for other time points.

	Time Since Contamination of Surface							
Surface	2 h	8 h	24 h	48 h	7 Days	28 Days	90 Days	180 Days
Glass	+	+	+	+	+	+	0, 3	N/A/+
Concrete	+	+	+	+	+	−	0, 3	N/A/−
Aluminium	+	+	+	+	+	1, 3	−	N/A/−
Hard Plastic	+	+	+	+	+	+	+	3, 0
Polythene	+	+	+	+	+	+	+	3, 0
Stainless steel	+	+	+	+	+	+	0, 3	−
Skin (Sap) *	+	N/A	N/A	N/A	N/A	N/A	N/A	N/A
Skin (Leaf) *	+	N/A	N/A	N/A	N/A	N/A	N/A	N/A
Gloves (Sap) *	+	N/A	N/A	N/A	N/A	N/A	N/A	N/A
Gloves (Leaf) *	+	N/A	N/A	N/A	N/A	N/A	N/A	N/A

**Table 3:** ELISA results of test plants swabbed from ToBRFV-contaminated hands after washing using different treatments. n, n represents the number of test plants positive out of 3 plants inoculated in each repetition.

	Treatment			
Length of Wash	Water Only	Water and Soap	Water and Medicated Hand Wash (Hibiscrub^®^)	Water and Medicated Hand Wash, Followed by Gel
30 s	3, 3	1, 2	1, 3	3, 2
1 min	0, 0	0, 0	2, 2	0, 0

**Table 4:** ELISA results of test plants swabbed from plastic trays contaminated with ToBRFV-infected sap before soaking in heated water, after soaking at different temperatures and post-soaking and spraying with Virkon (1% a.i.). n, n represents the number of test plants positive out of 3 plants inoculated in each repetition.

Temperature of Water	Pre-Treatment	5 min Soak in Water	After Soak + Virkon S
70 °C	3, 3	3, 3	0, 0
90 °C	3, 3	0, 0	0, 0

**Table 5:** ELISA results of test plants swabbed with ToBRFV-infected sap after soaking for 5 min at various temperatures. n, n represents the number of test plants positive out of 3 plants inoculated in each repetition.

Temperature (5 min Exposure)	No. of Plants Positive by ELISA	Comments
70 °C	3, 3	Many lesions seen on each test plant
80 °C	3, 3	Few lesions seen on each test plant
85 °C	2, 2	Only 1 lesion seen on each of 2 test plants
90 °C	0, 0	No lesions seen
95 °C	0, 0	No lesions seen

**Table 6:** Retention of infectivity of ToBRFV following exposure to disinfectant treatments for 60 min. n, n numbers represent the number of test plants positive out of 3 after each repetition of the experiment. Menno Florades applied as a foam (4% solution). Other disinfectants applied as a spray: Jet 5 1:125 dilution, sodium hypochlorite 0.04% a.i., Virkon S 1% a.i., Huwa San 12.5% a.i. and 3% a.i., TSOP 10% a.i., Unifect G 1:25 dilution and Virocid 1% dilution. After disinfection, swabs were taken and rubbed onto test plants. Test plants were tested by ELISA after 2 weeks to check infectivity.

	Product								
Surface	Menno Florades	Jet 5	Sodium Hypochlorite	Virkon S	Huwa-San 3% ai	Huwa-San 12.5% ai	TSOP	Unifect G	Virocid
Glass	0, 0	3, 2	1, 0	0, 0	2, 2	0, 0	1, 1	0, 0	0, 0
Concrete	1, 3	2, 0	0, 0	0, 2	1, 2	3, 3	2, 2	0, 0	0, 0
Aluminium	0, 0	2, 1	0, 0	0, 0	2, 2	0, 0	2, 2	0, 0	0, 0
Hard Plastic	0, 1	0, 1	0, 0	0, 0	2, 2	0, 0	2, 0	0, 0	0, 0
Polythene	0, 0	2, 0	1, 0	0, 0	0, 3	0, 0	2, 1	0, 0	0, 0
Stainless Steel	0, 0	3, 3	0, 2	0, 0	0, 2	0, 0	2, 2	0, 0	0, 0

**Table 7:** Determination of the minimum exposure time to prevent infection with selected products at a range of times. n, n numbers represent the number of test plants positive out of 3 after each repetition of the experiment.

	Product				
Surface	Unifect G 10 min	Virkon S 10 min	Virkon S 20 min	Menno Florades 16 h	Huwa-San 3% ai 16 h
Glass	0, 0	0, 2	0, 0	0, 0	1, 0
Concrete	0, 0	0, 0	3, 3	2, 2	1, 3
Aluminium	0, 0	2, 0	0, 0	0, 0	0, 1
Hard Plastic	0, 0	1, 0	0, 0	0, 0	2, 2
Polythene	0, 0	0, 0	0, 0	0, 0	0, 3
Stainless Steel	0, 0	2, 1	0, 0	0, 0	3, 2

**Table 8:** Molecular detection of ToBRFV post-disinfection treatment. Results presented in sub-columns headed “Ct Value” are real-time RT-PCR Ct values of swabs taken from surfaces contaminated with ToBRFV-infected sap, both pre-disinfection treatment and post-treatment (reps 1 and 2) 10 min after being sprayed with Virkon S or Unifect G. Data presented in the sub-columns headed “Inoculated test plants” are the interpretation of ELISA results from test plants inoculated with these swabs. n of 3 numbers represent the number of test plants positive out of 3.

	Virkon					Unifect G				
Surface	Ct Values			Inoculated Test Plants		Ct Values			Inoculated Test Plants	
	Pre-Treatment	Rep1	Rep2	Pre-Treatment	Post-Treatment	Pre-Treatment	Rep 1	Rep 2	Pre-Treatment	Post-Treatment
Glass	12	10	12	3 of 3	0 of 3	12	18	15	3 of 3	0 of 3
Concrete	12	12	11	3 of 3	0 of 3	10	16	15	3 of 3	0 of 3
Aluminium	11	12	11	3 of 3	2 of 3	11	15	14	3 of 3	0 of 3
Hard Plastic	12	12	11	3 of 3	1 of 3	13	15	16	3 of 3	0 of 3
Polythene	11	12	13	3 of 3	0 of 3	12	15	16	3 of 3	0 of 3
Stainless Steel	12	13	11	3 of 3	2 of 3	12	14	15	3 of 3	0 of 3

## Data Availability

Data are contained within the article and Supplementary Materials.

## Supplementary Materials

The following supporting information can be downloaded at: https://www.mdpi.com/article/10.3390/v15102076/s1, Table S1: ELISA results of test plants inoculated with swabs taken from hands (both gloved and bare skin) contaminated with ToBRFV infected sap or by rubbing infected leaves at different time periods.; Table S2: Retention of infectivity of ToBRFV following exposure to disinfectant treatments for 1 minute.
