# Supplementary material for: Tomato Brown Rugose Fruit Virus: Survival and Disinfection Efficacy on Common Glasshouse Surfaces
Source: Viruses. 2023 Oct 11;15(10):2076. doi: 10.3390/v15102076 (PMC10611295; doi:10.3390/v15102076)
Supplement: Supplementary file 1 [file viruses-15-02076-s001.zip › viruses-2637089-supplementary.pdf]

Supplementary data

Table S1. ELISA results of test plants inoculated with swabs taken from hands (both gloved and bare skin) contaminated with ToBRFV infected sap or by rubbing infected leaves at different time periods. Each result is from two repetitions of three plants per repetition. + = all plants positive in both repetitions.

	Time (minutes) after contamination with ToBRFV						
**Surface**	0	15	30	45	60	90	120
Skin (sap)	+	+	+	+	+	+	+
Gloves (sap)	+	+	+	+	+	+	+
Skin (leaves)	+	+	+	+	+	+	+
Gloves (leaves)	+	+	+	+	+	+	+

Table S2. Retention of infectivity of ToBRFV following exposure to disinfectant treatments for 1 minute. n, n numbers represent the number of test plants positive out of 3 after each repetition of the experiment. Menno Florades applied as a foam (4% solution). Other disinfectants applied as a spray; Jet 5 1:125 dilution, Sodium hypochlorite 0.04% a.i. & Virkon S 1% a.i.. After disinfection swabs were taken and rubbed onto test plants. Test plants were tested by ELISA after 2 weeks to confirm infectivity.

	Product			
**Surface**	**Menno Florades**	**Jet 5**	**Sodium Hypochlorite**	**Virkon S**
**Glass**	3, 3	3, 3	3, 3	3, 3
**Concrete**	3, 3	3, 3	3, 3	3, 3
**Aluminium**	3, 3	3, 3	3, 3	3, 3
**Hard Plastic**	3, 3	3, 3	3, 3	3, 3
**Polythene**	3, 3	3, 3	3, 3	3, 3
**Stainless Steel**	3, 3	3, 3	3, 3	3, 3
